# Potentially inappropriate prescribing and adverse drug reactions in the elderly: a population-based study

**DOI:** 10.1007/s00228-015-1950-8

**Published:** 2015-09-26

**Authors:** Khedidja Hedna, Katja M. Hakkarainen, Hanna Gyllensten, Anna K. Jönsson, Max Petzold, Staffan Hägg

**Affiliations:** Division of Drug Research, Department of Medical and Health Sciences, Linköping University, Linköping, Sweden; Nordic School of Public Health NHV, Gothenburg, Sweden; EPID Research, Espoo, Finland; Division of Insurance Medicine, Department of Clinical Neuroscience, Karolinska Institute, Stockholm, Sweden; Department of Clinical Pharmacology and Department of Medical and Health Sciences, Linköping University, Linköping, Sweden; Centre for Applied Biostatistics, University of Gothenburg, Gothenburg, Sweden; Futurum, Jönköping County Council, Jönköping, Sweden

**Keywords:** Inappropriate prescribing, Elderly, Adverse drug reactions, Retrospective study, Medical records, Registries

## Abstract

**Purpose:**

Potentially inappropriate prescriptions (PIPs) criteria are widely used for evaluating the quality of prescribing in elderly. However, there is limited evidence on their association with adverse drug reactions (ADRs) across healthcare settings. The study aimed to determine the prevalence of PIPs, defined by the Screening Tool of Older Persons’ potentially inappropriate Prescriptions (STOPP) criteria, in the Swedish elderly general population and to investigate the association between PIPs and occurrence of ADRs.

**Method:**

Persons ≥65 years old were identified from a random sample of 5025 adults drawn from the Swedish Total Population Register. A retrospective cohort study was conducted among 813 elderly with healthcare encounters in primary and specialised healthcare settings during a 3-month period in 2008. PIPs were identified from the Swedish Prescribed Drug Register, medical records and health administrative data. ADRs were independently identified by expert reviewers in a stepwise manner using the Howard criteria. Multivariable logistic regression examined the association between PIPs and ADRs.

**Results:**

Overall, 374 (46.0 %) persons had ≥1 PIPs and 159 (19.5 %) experienced ≥1 ADRs during the study period. In total, 29.8 % of all ADRs was considered caused by PIPs. Persons prescribed with PIPs had more than twofold increased odds of experiencing ADRs (OR 2.47; 95 % CI 1.65–3.69). PIPs were considered the cause of 60 % of ADRs affecting the vascular system, 50 % of ADRs affecting the nervous system and 62.5 % of ADRs resulting in falls.

**Conclusion:**

PIPs are common among the Swedish elderly and are associated with increased odds of experiencing ADRs. Thus, interventions to decrease PIPs may contribute to preventing ADRs, in particular ADRs associated with nervous and vascular disorders and falls.

**Electronic supplementary material:**

The online version of this article (doi:10.1007/s00228-015-1950-8) contains supplementary material, which is available to authorized users.

## Background

The rapid growth in the proportion of older population increases demands on healthcare systems worldwide [1]. Several factors contribute to the challenge of the care of the elderly, including comorbidities and chronic conditions often requiring multiple medications [2, 3], age-related physiological changes leading to increased sensitivity to drug effects [4] and limited evidence of drug effectiveness and safety in older and frail patients [5]. Previous studies have reported that up to 61 % of older patients in hospital settings develop adverse drug reactions (ADRs) [6], and approximately half of them are potentially preventable [7]. Potentially inappropriate prescriptions (PIPs) may be defined as “the prescriptions that introduce a significant risk of an adverse drug related event when there is evidence for an equally or more effective alternative medication” [8]. PIPs have been reported as an important cause of iatrogenic morbidity [9], mortality [10] and increased healthcare costs [11].

Explicit prescribing criteria have been developed to raise prescribers’ and other healthcare providers’ awareness about inappropriate prescribing and to improve the quality of prescribing in the elderly [12, 13]. The Screening Tool of Older Persons’ potentially inappropriate Prescriptions (STOPP), published in 2008 [14], has been endorsed by researchers in different jurisdictions, in Europe and elsewhere, for evaluating the quality of prescribing of elderly patients with multiple chronic conditions [15–18]. However, evidence of an association between PIPs identified by STOPP criteria and the occurrence of ADRs is limited [19] and mainly studied in hospital settings [20–22]. As the majority of healthcare contacts of the elderly occur in primary care [23], we aimed to determine the prevalence of PIPs, defined by STOPP criteria, in the Swedish elderly general population, including all care settings, and to study the association between PIPs and occurrence of ADRs.

## Methods

### Study design and study population

Individuals older than 65 years were identified from a random sample of 5025 adult residents in the County Council of Östergötland, drawn from the Total Population Register of Statistics Sweden [24]. A retrospective cohort study was conducted using the medical data of older patients, who had at least one healthcare encounter in primary or specialised care over a 3-month period in 2008.

### Data sources

Several data sources were linked using the personal identity number [25]. Data on prescribed medications were extracted from the Swedish Prescribed Drug Register (SPDR) [26]. The register includes dispensed prescribed drugs for outpatients, residential care and nursing homes, but excludes drugs administered in hospitals, and emergency drugs in residential care and nursing homes. Data on healthcare encounters were retrieved from the regional patient register (the Care Data Warehouse of Östergötland County), which includes administrative data on all inpatient and outpatient care provided in the county in all medical specialties and its coverage is considered full [27]. Based on the administrative care data, electronic medical records in all care units were reviewed during the study period. Sociodemographic characteristics were accessed from Statistics Sweden.

### Case assessment

#### Detection of PIPs

The STOPP criteria include 65 instances of common PIPs, including drug-drug and drug-disease interactions, unnecessary therapeutic duplication and drugs which can increase the risks of cognitive decline and falls in older patients [14]. Patient medical data, including medical histories, diagnoses and current medications, were recorded by one research pharmacist. Prescribed medications were identified from the SPDR through the Anatomical Therapeutic Chemical (ATC) classification system. PIPs were assessed during 6 months, including 3 months prior the study period. The research pharmacist referred to the research team in the event of uncertainty regarding interpretation of the clinical data and application of the STOPP criteria.

#### Detection of ADRs

An ADR was defined according to the World Health Organisation as “a response to a drug which is noxious and unintended, and which occurs at doses normally used in man…” [28]. ADRs were detected in a stepwise manner. All suspected ADRs occurring during the study period were initially analysed by other research pharmacists than the one who identified PIPs. They extracted information from the medical records for the 3-month study period, 9 months before and 3 months after. Used triggers included symptoms indicating worsening health status [29], common drug/adverse event combinations [29] and drug-drug interactions [30]. A clinical pharmacologist and a senior pharmacist independently assessed the causality between the prescribed medications and the suspected ADRs using Howard algorithm [31]. Conflicting assessments were solved by consensus. Suspected ADRs with at least possible causality were considered ADRs. The seriousness of ADRs was assessed [32]. Finally, PIPs with causal contribution to the identified ADRs were considered.

### Statistical analysis

The baseline characteristics of the study population included the number of prescribed medications, the level of healthcare use (defined by Diagnosis Related Group (DRG) weights [33]) 3 months prior to the study period and the most common morbidities. We estimated the prevalence of individuals with at least one PIP, with elderly individuals who had a healthcare encounter during the study period as the denominator. The most common PIPs (those occurring in at least ten individuals) and the proportion causing ADRs was reported.

The 3-month prevalence of individuals with ADRs and the proportion of ADRs considered as caused by PIPs were calculated. The ten most common organ system disorders and symptoms, as defined by the Medical Dictionary for Regulatory Activities (MedDRA) [34], were reported, and the proportion considered as caused by PIPs was calculated. Serious ADRs judged to be caused by PIPs were described.

The association between PIPs and ADRs was investigated with a multivariable logistic regression. The results were adjusted for age (65–74, 75–84, ≥85 years), sex, number of dispensed prescribed medications (0, 1, 2–5, 6–9, ≥10), level of healthcare use and use of multidose drug dispensing [35]. Data analysis was performed using Stata version 11.1 (StataCorp, TX). *P* value of less than 0.05 was considered statistically significant.

Finally, a sensitivity analysis was performed without the 12 criteria that are excluded from the updated STOPP version (November 2014) [36].

### Ethical consideration

The study was approved by the Regional Ethical Review Board in Gothenburg (no: 644-2008) according to the Swedish regulation. Informed consent of participants was not required as the retrospective study design did not affect the healthcare of included patients. Statistics Sweden replaced the personal identity numbers by a random serial number after the final data linkage and data were analysed anonymously.

## Results

Data were collected from 813 elderly. The main characteristics of the study population are summarised in Table 1. The median age was 75.0 years (range 65–98 years). In total, 66.7 % had encounters exclusively in primary care, and 7.3 % was hospitalised 3 months prior to the study period. Overall, 25.2 % of the study population was prescribed 6 to 9 medications and 12.0 % ≥10 medications.

**Table 1:** Study population characteristics (*n* = 813)

Characteristics	*n* (%)
Age (years)	
Median (range)	75.0 (65–98)
65–74	401 (49.3)
75–84	289 (35.6)
≥85	123 (15.1)
Sex	
Female	458 (56.3)
Dispensed prescribed medications^a^	
Median (range)	4.0 (0–20)
0	90 (11.1)
1	72 (8.9)
2–5	384 (42.8)
6–9	205 (25.2)
≥10	98 (12.0)
Patients prescribed cardiovascular medications^b^	595 (73.2)
Patients prescribed psychotropic medications^c^	278 (34.2)
Use of multiple drug dispensing	85 (10.4)
Level of healthcare use^d^	
Primary care	542 (66.7)
Specialised (outpatient or inpatient) care	271 (33.3)
Hospitalisation	59 (7.3)
Morbidities	
Hypertension	358 (44.0)
Diabetes	158 (19.4)
Ischemic heart disease	147 (18.1)
Mental and behavioural disorders	140 (17.2)
Heart failure	85 (10.5)
Osteoarthritis	83 (10.2)
Chronic obstructive pulmonary disease	68 (8.4)
Dementia	28 (3.4)

^a^Three months prior to the study period

^b^Anatomical Therapeutic Chemical code C

^c^Anatomical Therapeutic Chemical code N05 and N06

^d^Defined by DRG weights

We found 607 PIPs prescribed to 374 persons (46.0 %) (Table 2). The prevalence of PIPs was 42.8 % among those with exclusively primary healthcare contacts, 52.4 % among those with specialised healthcare and 66.1 % among the elderly who were hospitalised at least once during the 3-month study period. Multivariable regression analysis showed that persons prescribed PIPs had more than twofold increased odds to experience ADRs (odds ratio (OR) 2.47, 95 % confidence interval (CI) 1.65–3.69); *p* < 0.001), compared to that in persons without PIPs.

**Table 2:** Number of potentially inappropriate prescriptions in persons (*n* = 813)

Number of PIPs/person	*n* (%)
Mean (range)	0.76 (0–9)
Number of persons with ≥1 PIPs	374 (46.0)
1	221 (27.2)
2	102 (12.6)
3	28 (3.4)
≥4	23 (2.8)
PIPs in persons using only primary care	226 (42.8)
PIPs in persons using specialised healthcare	148 (52.4)
PIPs in hospitalised persons	33 (66.1)

*PIP* potentially inappropriate prescriptions

The most common PIPs are described in Table 3. In total, 10.5 % of PIPs caused ADRs (Table 3). The percentage of PIPs considered causing ADRs was the highest for vasodilators in persons with persistent postural hypotension (92.3 % of PIPs causing ADRs), prolonged use of neuroleptics (46.2 %), first-generation antihistamines (25.0 %) and benzodiazepines (23.3 %) in those prone to fall.

**Table 3:** Most common potentially inappropriate prescriptions

Criterion	*n* (%)	*n* (%) causing ADRs
Aspirin with no history of coronary, cerebral or peripheral arterial symptoms or occlusive arterial event	154 (18.9)	7 (6.5)
Benzodiazepines in those prone to fall	43 (5.4)	10 (23.3)
NSAID with moderate-severe hypertension	41 (5.0)	1 (2.4)
Long-term long-acting benzodiazepines	37 (4.6)	3 (8.1)
Prolonged use (>1 week) of first generation antihistamines	28 (3.4)	7 (25.0)
Use of long-term powerful opiates as first-line therapy for mild-moderate pain	27 (3.3)	0 (0)
Long-term opiates in those with recurrent falls	26 (3.2)	6 (23.1)
Long-term (i.e. >1 month) neuroleptics as long-term hypnotics	26 (3.2)	12 (46.2)
Oestrogens without progestogen in patients with intact uterus	23 (2.8)	0 (0)
Long-term corticosteroids (>3 months) as monotherapy for rheumatoid arthritis or osteoarthritis	19 (2.3)	2 (10.5)
Aspirin at dose >150 mg/day	17 (2.1)	0
Long-term use of NSAID (>3 months) for relief of mild joint pain in osteoarthritis	14 (1.7)	0
Vasodilator drugs known to cause hypotension in those with persistent postural hypotension	13 (1.6)	12 (92.3)
Systemic corticosteroids instead of inhaled corticosteroids for maintenance therapy in moderate–severe COPD	12 (1.5)	0
First-generation antihistamines in those prone to fall	11 (1.4)	1 (9.1)
Neuroleptic drugs in those prone to fall	10 (1.2)	0
Total	607 PIPs (374 persons)	64 (10.5)

*ADR* adverse drug reaction, *NSAID* non-steroidal anti-inflammatory drugs, *COPD* chronic obstructive pulmonary disease

**Fig. 1:**
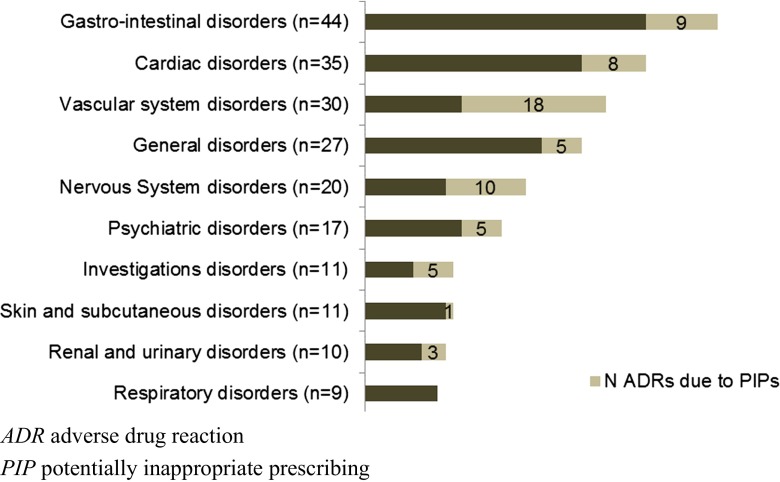
Organs affected by adverse drug reactions and the proportion considered by potentially inappropriate prescriptions

**Fig. 2:**
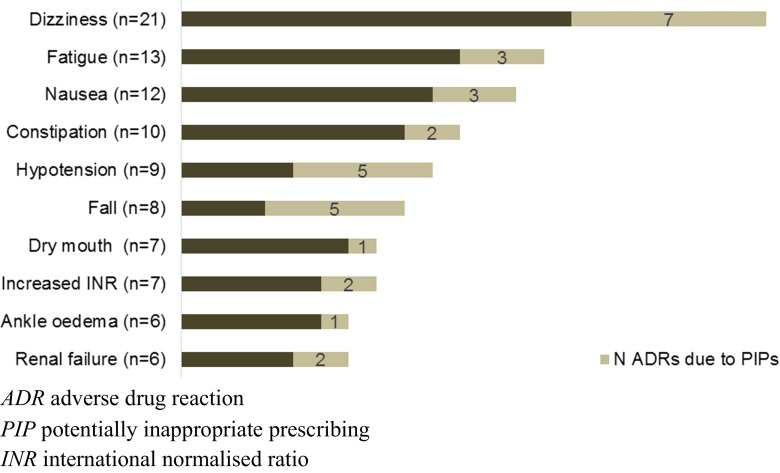
The most common symptoms of adverse drug reactions and the proportion caused by potentially inappropriate prescriptions

Overall, 245 ADRs were identified in 159 persons (19.6 %), of which 73 were considered as caused by PIPs (29.8 % of all ADRs). PIPs were considered the cause of a high percentage of ADRs affecting the vascular and nervous systems (60.0 and 50.0 %, respectively) (Fig. 1). Moreover, 62.5 % of ADRs resulting in falls were considered as caused by inappropriate use of benzodiazepines (Fig. 2).

Twelve serious ADRs were identified; among them, eight were considered caused by PIPs mainly by antipsychotic and non-steroidal anti-inflammatory drugs (Supplement 1). One death was judged to be caused by the long-term use of nitrazepam.

### Sensitivity analysis

When the analyses were limited to the 53 criteria included in the updated STOPP version [36], 270 (33.2 %) elderly persons had at least one PIP, 24.9 % of ADRs were considered as caused by PIPs, and PIPs were significantly associated with the occurrence of ADRs (OR 2.57, 95 % CI 1.75–3.77, *p* < 0.001).

## Discussion

Nearly half of patients across care settings were prescribed with one or more PIPs during a 3-month period. Moreover, one third of ADRs were considered caused by PIPs, and elderly who were prescribed with PIPs had over twofold increased odds to experience ADRs.

Our findings indicate that PIPs are common among patients in both primary and specialised healthcare. The prevalence of PIPs in studies using the STOPP criteria has ranged between 21 and 79 %, depending on the study setting and design [19]. In accordance with a Spanish study [37], our PIP prevalence was higher in specialised care compared to that in primary care. Persons using more advanced care probably have more complex comorbidities, which have been associated with PIPs [38]. However, elderly in specialised care may not be representative of the elderly population as a whole. As the majority of elderly use mainly primary care, the understanding of PIPs in primary care must be improved. Furthermore, the validity of detecting PIPs among the elderly in the general population should be investigated.

The most frequent PIPs in our study partially differed from common PIPs in hospital-based studies [19, 20, 39, 40]. Similarly to studies conducted in hospital settings [19], the long-term use of long-acting benzodiazepines and medications increasing the risk of fall (such as benzodiazepines, opiates and first-generation antihistamines) were among the most common PIPs in our study. However, while some PIPs, such as proton-pump inhibitors for peptic ulcer at full therapeutic dosage for >8 weeks, were frequently reported among the most common PIPs [20, 39, 40], they were rarely reported in our study. Nonetheless, some frequently reported PIPs in our study, such as the inappropriate prescribing of aspirin, were rarely reported in studies conducted exclusively in hospital settings [40]. The variation in the most common PIPs between studies is, in addition to care settings, probably explained by differing prescribing patterns and clinical practice guidelines, prescribing regulation, population characteristics and disease burden [40–42]. For example, the higher frequency of inappropriate prescribing of aspirin in our study compared to that in previous studies may be due to the commonness of aspirin as an over-the-counter medication in other countries [43], underestimating its inappropriate use in studies using exclusively prescription data.

We found a large part of PIPs associated with ADRs involving medications that increase the risk of falls, such as benzodiazepines, opiates and vasodilators, which indicate that decreasing PIPs could contribute towards fall prevention. Though falls are considered multifactorial [44], our study found a high percentage of falls (or associated symptoms such as dizziness and orthostatic hypotension) caused by PIPs and, thus, considered as potentially preventable. Patients may be unable to recognise ADRs, such as hypotension or dizziness, or not report them to their healthcare givers, increasing the risk of experiencing falls as ADRs, if the medication regimen is not adjusted [45]. Medication review by prescribers and other healthcare professionals, including a comprehensive falls assessment, could decrease such PIPs, as shown by a prescribing education programme for primary care physicians, which significantly reduced the risk of fall among elderly patients [46]. Vasodilators appear particularly strongly associated with falls or associated symptoms; as in our study, the percentage of PIPs associated with ADRs was the highest for vasodilators in those with persistent postural hypotension. Safety issues of medications with repeat prescribing including vasodilators have also been previously warranted [47].

Our study including all care settings found elderly with PIPs having significantly increased odds of experiencing ADRs, demonstrating that PIPs cause potentially preventable morbidity across care settings. Most ADRs due to PIPs in our study were non-serious, while some previous studies have mainly focused on the associations between PIPs and serious adverse outcomes, such as hospitalisation and death [10, 48]. However, even mild ADRs are important to consider as they are associated with lower quality of life, may increase the visits to general practitioners and cause prescribing cascades to treat symptoms of unrecognised ADRs [49]. Although we identified few serious ADRs due to PIPs across care settings, the ADRs included one fatal case due to long-term use of long-acting benzodiazepines, highly recognised to be inappropriate in the elderly. This suggests that improving the quality of prescribing could also prevent some fatal ADRs.

The association between PIPs and ADRs remained significant after limiting the analysis exclusively to the STOPP criteria included in the updated version. Thus, excluding certain criteria in the updated version appears relevant. We should emphasise, however, that the updated version was extended to 76 criteria, among them 23 criteria not listed in the first version, including some general ones as “Any drug prescribed without an evidence-based clinical indication or beyond the recommended duration”. Moreover, the new version considers the use of benzodiazepines for more than 4 weeks as inappropriate, which was found in 23.2 % of our study population. Therefore, our estimation of PIPs prevalence may not be an overestimation, although the most common inappropriate drugs would differ between the two versions.

### Strengths and limitations

Our study is the first that investigated the association between PIPs according to STOPP criteria and ADRs among a representative sample of the general elderly population. Nevertheless, our findings should be interpreted with some limitations in mind. STOPP criteria are widely used to evaluate the quality of prescribing in the elderly. However, in some cases, medications classified as potentially inappropriate may be appropriate considering individual patient’s health condition. Yet, the assessment of PIPs’ causality or contribution to the detected ADR was based on a validated causality assessment algorithm [31]. The SPDR includes prescriptions in outpatients and nursing homes, and we could not include prescriptions during hospitalisations and emergency drugs in nursing homes and specialised care. However, only 7 % of the study population had short hospitalisation episodes during the study period, which also raises the question about the generalisability of studies conducted only in hospital settings. We were also unable to stratify our regression analysis by the type of residence, due to unavailability of these data. Although previous studies have found significant differences in PIPs by type of residence [37], the potentially small number of elderly living in nursing homes [50] would, however, limit interpreting such stratified analysis.

The study was conducted across different healthcare organisations, with different quality and quantity of medical record data. Some PIP criteria were impossible to evaluate in cases with insufficient clinical information and history (e.g. information about an intact uterus). However, our method of assessment of cases, based on symptoms, biological and clinical data, allowed the detection of ADRs that were not recognised or reported as such in the medical records. However, symptoms of ADRs not communicated by patients or not recorded by care providers in the medical records could not be detected in this study.

While we adjusted our regression model with known factors associated with PIPs and ADRs, some confounders might have been undetected. Further, we purposefully did not consider the new criteria in the sensitivity analysis as they are based on new recommendations that may be irrelevant to apply to prescriptions in 2008.

### Implications

The STOPP criteria may be a useful tool for screening and identifying potential ADRs in older people across healthcare settings. While previous studies have found PIPs detected with the STOPP criteria significantly associated with ADRs among hospitalised patients [21], our study extend the evidence on their use to detect ADRs across healthcare settings. However, the applicability of the STOPP criteria in clinical practice and community pharmacy needs to be established. There is an ongoing study to integrate the STOPP criteria in an electronic automated format [51]. Nevertheless, integrating the STOPP criteria in the medication use reviews in community pharmacies and general practices requires an access to complete patient clinical data and a learning time to familiarise with them [19].

Based on our results, the STOPP criteria seem to be particularly sensitive to detect nervous and vascular disorders and falls. However, a better understanding of the two thirds of ADRs due to medications not listed in the PIPs criteria, including over-the-counter medications is needed, as they have been associated with increased morbidity and hospitalisations [52].

Reducing both PIPs and ADRs among the elderly will require system interventions to routinely assess drug appropriateness, effectiveness, safety and adherence, while balancing the risk of underuse of beneficial medications [53]. Moreover, there is a need for valid patient-centred prescribing evaluation tools across care settings to track patients’ perceived adverse outcomes and to engage them in monitoring their medications [53]. Improving the quality of prescribing requires a collaboration of prescribers and other healthcare professionals and a better continuity of care of patients with chronic conditions [54].

## Conclusion

In conclusion, PIPs are common among the Swedish elderly and are associated with twofold increased odds to experience ADRs. The PIP criteria defined by STOPP are not a substitute for clinical assessment and judgement, but they may encourage clinicians to consider medications as a possible cause of adverse health outcomes, in particular nervous system and vascular disorders and falls. Thus, interventions to decrease PIPs may contribute to preventing ADRs among the elderly.

## Electronic supplementary material

ESM 1 (DOCX 16 kb)
