# Supplementary material for: Potentially inappropriate prescribing and adverse drug reactions in the elderly: a population-based study
Source: Eur J Clin Pharmacol. 2015 Sep 26;71(12):1525–33. doi: 10.1007/s00228-015-1950-8 (PMC4643104; doi:10.1007/s00228-015-1950-8)
Supplement: Supplementary file 1 — (DOCX 16 kb) [file 228_2015_1950_MOESM1_ESM.docx]

**Supplement 1. Serious adverse drug reactions among the elderly caused by potentially inappropriate prescriptions**

- Sudden death associated with long-term use of nitrazepam.
- Recurrent falls and severe confusion state associated with long-term use of morphine (1 case) or benzodiazepines (1 case) in a person with a previous history of falls.
- Severe hypotension associated with prescription of antihypertensive medications with previous episodes of orthostatic hypotension.
- Gastric ulcer perforation associated with long-term use of diclofenac for relief of mild pain in osteoarthritis.
- Severe headache associated with prolonged use of hydroxyzine.
- Severe renal failure associated with chronic use of naproxen with previous diagnosis of reduced renal function (2 cases).
